# Selecting Optimal Housekeeping Genes for RT-qPCR in Endometrial Cancer Studies: A Narrative Review

**DOI:** 10.3390/ijms26178610

**Published:** 2025-09-04

**Authors:** Maciej Jóźwik, Iwona Sidorkiewicz, Joanna Wojtkiewicz, Stanisław Sulkowski, Andrzej Semczuk, Marcin Jóźwik

**Affiliations:** 1 Department of Gynecology and Gynecologic Oncology, Medical University of Białystok, 15-276 Białystok, Poland; 2 Clinical Research Support Centre, Medical University of Białystok, 15-276 Białystok, Poland; iwona.sidorkiewicz@umb.edu.pl; 3 Department of Human Physiology and Pathophysiology, Faculty of Medicine, Collegium Medicum, University of Warmia and Mazury in Olsztyn, 10-082 Olsztyn, Poland; joanna.wojtkiewicz@uwm.edu.pl; 4 Department of General Pathomorphology, Medical University of Białystok, 15-269 Białystok, Poland; stanislaw.sulkowski@umb.edu.pl; 5 IInd Department of Gynecological Surgery and Gynecological Oncology, Lublin Medical University, 20-090 Lublin, Poland; andrzej.semczuk@umlub.pl; 6 Department of Gynecology and Obstetrics, Collegium Medicum, University of Warmia and Mazury in Olsztyn, 10-045 Olsztyn, Poland; marcin.jozwik@uwm.edu.pl

**Keywords:** endometrial cancer, endometrium, genes, housekeeping, polymerase chain reaction

## Abstract

Detailed analysis of gene expression by real time-quantitative polymerase chain reaction (RT-qPCR) has become a widespread method. To normalize the expression of target genes, this approach relies on constitutively expressed internal controls known as housekeeping genes (HKGs). Their proper selection is a critically important methodological step, since all the studied gene expression will be recalculated based on HKG expression. This concise review aims to discuss the selection of HKGs for endometrial cancer (EC) studies. We draw attention to the fact that the commonly used gene glyceraldehyde-3-phosphate dehydrogenase (*GAPDH*) is unsuitable as a HKG for research on the normal endometrium, EC, as well as many other tissues. In contrast, accumulating evidence suggests that GAPDH is a pan-cancer marker and an EC marker. Work on *GAPDH* overexpression in EC in relation to overall and relapse-free survival is lacking. Both original research and overviews indicate that at least two HKGs should be used for target gene expression recalculations, a rarely applied technical aspect of final data processing. The insufficiently careful selection in many studies of only one HKG, e.g., *GAPDH*, can be held responsible for broad discrepancies in published results obtained by this RT-qPCR technique. We provide an account of the discrepancies reported for sex hormone receptors expression in EC. Achieving consensus on the selection and validation of HKGs for research on this cancer is of crucial importance. Ideally, this trusted gene combination should be universal for any EC histotype and grade, irrespective of the final anatomopathological result.

## 1. Introduction

Over the last quarter-century, detailed analysis of gene expression by real time-quantitative polymerase chain reaction (RT-qPCR) has become a widely used method, providing insights into molecular mechanisms of disease. Gene expression signatures have been instrumental in defining the molecular phenotypes of cells, tissues, and patient samples. In order to normalize the expression of target genes, constitutively expressed internal controls known as housekeeping genes (HKGs) are applied. Specifically, HKGs help to normalize mRNA levels between different samples, providing relative quantification. The proper selection of HKGs is a critically important step in accurately determining the expression of genes under study. This choice is based on the assumption of their inherent stability [1].

In 1965, Watson et al. defined HKGs as genes that are “always expressed” in every tissue to maintain cellular functions [2]. Another working concept put forth in 2000 has been that HKGs are “those genes critical to the activities that must be carried out for successful completion of the cell cycle. ” Furthermore, “they are genes that play a key role in the maintenance of every cell.” [3]. That is why Warrington et al. proposed re-naming them to “cell maintenance” or simply “maintenance” genes [3]. As such, they are presumed to produce the minimally essential transcripts necessary for normal cellular physiology [4]. Another description states that HKGs constitute a basal transcriptome for the maintenance of basic cellular functions [5]. In line with this, an analysis of the patterns of sequence evolution of 1581 human-mouse orthologous gene pairs compared HKGs with tissue-specific genes and concluded that HKGs, on average, evolve more slowly [6]. A verification of Serial Analysis of Gene Expression (SAGE) data for 14 human tissues indicated that HKGs show strong clustering on chromosomes, in contrast to tissue-specific genes that do not cluster as a rule [7]. Further, HKGs were found to be phylogenetically older than tissue-specific genes [8]. Some reports have stated that HKGs are less compact (i.e., all their length parameters were found to be longer) than tissue-specific genes [8], whilst other reports found that all parts of HKGs were, on average, shorter than other genes [9]. According to the Warrington study, human tissues share a set of 535 transcripts that are turned on early in fetal development and stay on throughout adulthood [3]; Hsiao et al. gave the figure of 451 HKGs present across 19 distinct human tissues [10], and further research reported on 575 HKGs across 47 different human tissues and cell lines [9]. However, a later estimate based on nearly 8 million Expressed Sequence Tags (ESTs) from 4 026 RNA tissue and organ samples indicated a number of human HKGs ranging from 3 140 to 6 909 [5]. In a word, there is a wide array of putative HKGs to verify and choose from.

The technical step of RT-qPCR for correcting for sample-to-sample variation is to amplify, simultaneously with the target, a cellular RNA that serves as an internal reference against which other RNA values can be normalized [1,11]. By doing so, expression levels of the gene(s) under study will be controlled for differences in cellular input, RNA quality, and reverse transcription efficiency [12]. Yet, the question of what constitutes an appropriate standard arises. Ideally, the internal standard should be expressed at a constant level among the different tissues of an organism at all stages of development and should be unaffected by the experimental treatment. In addition, an endogenous control gene should also be expressed at roughly the same level as the RNA under study [1]. Optimally, HKGs should be adequately expressed in the target tissue and demonstrate minimal variability and high stability in both health and disease [13,14]. However, to what extent this stability is maintained in disease states is controversial and is believed by many to be rather varied [14]. Some authors even think that it is unlikely that an ideal gene serving as an internal control exists, since biological systems are dynamic and constantly responding to their environment. Ergo, the most appropriate internal control would be one that has the least variation in its expression under various experimental conditions and in different tissue types [13].

In the past, studies on gene expression in gynecological tissues have conventionally used glyceraldehyde-3-phosphate dehydrogenase (*GAPDH*), 18S ribosomal RNA (18S rRNA), or β-actin (*ACTB*) gene as the HKG [14]. Briefly, 18S rRNA is the structural RNA for the small component of cytoplasmic ribosomes and is thus one of the basic components of eukaryotic cells. The other two frequently used normalizers are non-ribosomal. ACTB is one of six different isoforms of the protein identified in humans, i.e., one of the two nonmuscle cytoskeletal actins. As such, its mRNA is expressed at moderately abundant levels in most cell types and encodes a ubiquitous cytoskeleton protein [1]. As indicated by its name, GAPDH (EC 1.2.1.12) catalyzes the sixth step in the glycolytic breakdown of glucose in the cytosol, namely, the conversion of glyceraldehyde 3-phosphate to D-glycerate 1,3-bisphosphate. This oxidative phosphorylation is a reversible reaction. However, besides taking part in glycolysis, GAPDH is also a multifunctional moonlighting protein (i.e., a protein which exhibits numerous activities in different subcellular locales, apart from its initially well-characterized function) involved in an increasing number of newly discovered intracellular activities. Authoritative reviews indicate that membrane-bound GAPDH takes part in membrane fusion, endocytosis, and iron transport. Cytoplasmic GAPDH regulates mRNA stability and is required for endoplasmic reticulum-to-Golgi trafficking, whereas nuclear GAPDH participates in apoptosis, transcriptional gene regulation, the maintenance of DNA integrity, including DNA replication and repair, as well as the exportation of transfer RNA to cytosol. Furthermore, it is involved in intermembrane trafficking, synaptic transmission, microtubule bundling, heme metabolism, and the immune response [15,16,17]. Alarmingly, this pleiotropic enzyme has been implicated in many oncogenic roles, such as tumor survival, hypoxic tumor cell growth, tumor angiogenesis, control of tumor cell gene expression, and posttranscriptional regulation of tumor cell mRNA [16,17]. Recent reviews explain both GAPDH’s direct and posttranslational oncogenic involvements [17,18]. These are facilitated not only by the protein’s abundance in the cell but also by its ability to serve a variety of cellular functions within membrane, cytoplasmic, and nuclear compartments [19].

## 2. A Critique of Glyceraldehyde-3-Phosphate Dehydrogenase as a Housekeeping Gene

Criticism of the use of *GAPDH* as a HKG has been accumulating from many directions. The expert review by Bustin noted that while *ACTB* may still be advocated as a quantitative reference for RT-qPCR assays in selected, verified contexts in spite of the evidence that its levels of transcription can vary widely in response to experimental manipulation (as well as primers commonly used for detecting *ACTB* mRNA can amplify DNA), *GAPDH* cannot [1]. More importantly, that author indicated, with abundant literature citations, that *GAPDH* levels vary significantly among different individuals, among samples taken from the same individual at different time points, with developmental stage, during the cell cycle, and after the addition of the tumor promoters 12-*O*-tetradecanoyl-phorbol-13-acetate, dexamethasone, and carbon tetrachloride. *GAPDH* transcription is induced by insulin, growth hormone, vitamin D, oxidative stress, apoptosis, tumor protein p53, and nitric oxide, among others. In contrast, fasting and retinoic acid downregulate *GAPDH* transcription [1,18]. A large study on 72 pathologically normal human tissues in 1595 individual samples from 618 donors confirmed substantial between-tissue variations in the levels of *GAPDH* mRNA expression and a lack of effect of age and sex on these levels [20]. Taking all these important findings together, the use of *GAPDH* to normalize RNA levels in experiments using mRNA from different individuals has been strongly discouraged due to its overt inaccuracy [1].

Further, in 2001, Hsiao et al. [10] reported a compendium on gene expression in 19 different healthy tissue types obtained from adult men and women. With the use of oligonucleotide microarrays, they analyzed the expression of 7070 unique sequences to detect some similarities and, more frequently, striking quantitative differences among tissues, even for genes expressed constitutively. Of note, both *ACTB* and *GAPDH*, commonly assumed to have constant expression levels, were among the most variable genes [10]. Thus, *GAPDH* should not be a HKG choice for comparative studies across normal tissues and organs.

In a laboratory investigation on the reliability of exogenous versus endogenous PCR standards, *GAPDH* was found to be an (excessively) abundant endogenous standard and was thus regarded as being unreliable for quantitative or semi-quantitative PCR [21].

The assumption that HKGs are equally stable under all conditions must therefore be questioned [14,22]. For example, a comparative study on a serum-stimulated and serum-starved line of fibroblasts transfected with an inducible chimeric gene indicated how conditions of nourishment and starvation affect the expression of HKGs: 18S rRNA and *β2-microglobulin* (*B2M*) turned out to be suitable internal control genes in quantitative serum-stimulation studies, whereas *GAPDH* and *ACTB* did not. Consequently, the authors postulated that internal control genes need to be properly validated when designing quantitative gene expression studies [23]. In another comparison of RNA levels of potential HKGs under hypoxic and normoxic conditions, it was found that cellular expression levels of *GAPDH*, *ACTB,* and peptidylpropyl isomerase A (cyclophilin A; *PPIA*) genes varied widely with hypoxia, whilst levels of 28S rRNA were constant and independent of oxygen tension. *GAPDH* transcription was increased in hypoxia in particular, and since it correlated in prostate cancer cell lines with the upregulation of hypoxia-inducible factor-1α protein levels, *GAPDH* was considered particularly unfavorable as an endogenous control gene in hypoxic conditions [24]. Similarly, when the effect of varied hypoxic conditions was evaluated in two cancer and two normal cell lines, a tremendous variation in the expression of the majority of 10 frequently used normalizers was found, again with *GAPDH* being one of the most variable HKGs [25]. Particularly convincing evidence of how candidate HKGs can vary in a changing clinical context was provided by the study by Waxman and Wurmbach, who analyzed the differential expression of six genes, including *GAPDH* and *ACTB*, over eight consecutive stages of hepatitis C virus-induced hepatocellular carcinoma. The most constantly expressed genes across that investigation were *RPL41* (Ribosomal protein L41) and *SFRS4* (serine and arginine rich splicing factor 4), whose combination was found to be the most reliable for normalization in hepatocellular carcinoma. In contrast, *GAPDH* expression was strongly upregulated in advanced and very advanced tumors, in some samples up to seven-fold [26]. Many other clinical situations affecting *GAPDH* expression are listed in an early review [27].

Indeed, a solid body of evidence demonstrates with diverse laboratory techniques that *GAPDH* mRNA levels vary among normal cells and different malignancies, cancer cell lines, as well as cancer tissue biopsies [22,25,26,28,29,30,31,32,33,34,35,36,37,38,39,40,41,42,43,44,45,46,47,48,49,50]. Clearly, *GAPDH* expression is increased in malignant cells. In a study where median *GAPDH* expression in non-small cell lung cancer was slightly less than in control healthy tissue, a substantial median fold change (>2.4)—or lack of stability—of expression levels between healthy and tumor tissue was noted [51]. Similarly, median *GAPDH* expression in esophageal squamous cell carcinoma was lower than in adjacent normal esophageal mucosa; however, excessive variability excluded it from HKGs [52]. Likewise, a lack of stability in *GAPDH* levels between several human cancers and their respective control tissues was found in a Belgian study [53]. Not surprisingly, the upregulated *GAPDH* expression in oncogene-transformed fibroblasts has been shown to correlate with oncogene expression rather than cell growth fraction [32], in keeping with the enzyme’s many oncogenic roles. This is because malignant tumors rely largely, if not entirely, on the Warburg effect, where the role of GAPDH in aerobic glycolysis is basic [54,55]. In the analysis of the National Institutes of Health public database ‘dbEST’ for the expression of genes and ESTs, all 10 enzymes of glycolysis were found to be ubiquitously overexpressed in many human malignancies, including cervical, uterine, and ovarian cancer, with *GAPDH* overexpression at the forefront [43]. That study confirmed and extended previous results on primary colorectal cancer and secondary hepatic metastases, where both *PGK* (phosphoglycerate kinase) and *GAPDH* were significantly upregulated, in the metastases in particular [22,44]. The highly interesting finding that *GAPDH* expression is more elevated in the matching liver metastases than in the original cancer tissue was later confirmed by Chinese authors [46] and for cutaneous melanoma metastases by Spanish authors [48], pointing to the little explored role of *GAPDH* upregulation in metastasis formation and spread.

The involvement of GAPDH in cancer biology is actually so significant that, based on three independent, publicly available cohorts creating a large microarray database on non-small cell lung cancer, an American team performed a gene expression correlation analysis identifying many genes whose upregulation in the tumor closely (i.e., highly significantly) correlated with *GAPDH* overexpression. The authors were able to assign these genes to two classes: (1) cell cycle-related genes (accordingly designated as *GAPDH* Associated Cell Cycle genes (GACC)); and (2) metabolism-related genes. Notably, the expression of each gene in the glycolysis pathway, including *GADPH*, was found to be significantly upregulated in this cancer [47].

With regard to endometrial cancer (EC), particularly robust evidence comes from explorations of large electronic databases. In data extracted from three cancer-related Gene Expression Omnibus datasets, *GAPDH* expression in EC was found to be highly statistically increased (*p* < 0.001) when compared with normal endometrium [50]. In line with this, based on the data extracted from The Cancer Genome Atlas (TCGA) of the National Cancer Institute, USA, *GAPDH* mRNA expression in EC tumors is highly statistically increased (*p* < 0.001) compared with adjacent normal tissue [49]. *GAPDH* overexpression in other tumors has been unanimously associated with poor overall and relapse-free survival [39,47,49,50].

Corroborating much of the above information with several bioinformatic tools, Shen et al. postulated that *GAPDH* is not only unsuitable as an internal reference gene for most cancer research, whether by RNA or protein analyses, but also, it may be a promising biomarker of pan-cancer prognosis [49]. Let us underline the important notion that *GAPDH* may be a particularly poor normalizer of RT-qPCR studies on cancer metastases [22,44,46,48].

Comparative research on uterine and omental artery endothelial cells from pregnant and non-pregnant ewes suggested that *GAPDH* transcript is not likely to be a HKG standard for any pregnancy-related condition either. Pregnancy was associated with a 4.5-fold increase in *GAPDH* mRNA levels and 1.6-fold increase in GAPDH protein expression [56]. Another animal study found that substantial changes in the composition of RNA species, and especially a decrease of total RNA, occur through gestation, limiting the use of HKGs to normalize RT-qPCR data, especially when absolute quantification is required [57].

Works reporting *GAPDH* as a reliable HKG are rare; we were able to trace only a few such studies. The enzyme’s transcript levels in human diploid fibroblasts were found to be unaffected across four different treatment groups: young cells of passage 4, senescent cells of passage 30, cells subjected to H_2_O_2_-induced oxidative stress, and cells treated with γ-tocotrienol [58]. In the Finnegan study on non-Hodgkin’s lymphoma, *GADPH* expression was judged to be unacceptable for a HKG, even though it was the least unstable among the tested genes [59]. Contrary to later research [48], the levels of *GAPDH* expression in the Seykora study were found to be similar in both nevi and melanomas; however, that investigation did not include normal skin controls [60].

## 3. Housekeeping Gene(s) for Studies of Normal Endometrium

When sampling the endometrium or other tissues from women of reproductive age (usually as a reference, or so-called ‘control’), our team does its best to obtain tissues on Day 2 after the cessation of menstruation, i.e., in the mid-follicular phase [61]. By doing so, we aim to minimize the effect of changes in gene expression during the menstrual cycle, or at least control for this internal variability. In the normal endometrium of women of reproductive age, significant changes in gene expression relative to particular phases of the menstrual cycle have been reported [62,63].

Regarding suitable HKGs, although the compendium of gene expression in normal human tissues did analyze endometrial and myometrial specimens and aimed to report tissue-selective reference genes, no particular recommendation came from that study [10]. The Sadek study demonstrated that *GAPDH* is relatively unsuitable for the normalization of endometrial tissue from polycystic ovary syndrome and normal women of reproductive age, whereas *YWHAZ* (tyrosine 3-monooxygenase/tryptophan 5-monooxygenase activation protein zeta), *CYC1* (cytochrome c-1), and *ACTB* showed acceptably small variation in expression across these two conditions [14]. Gebeh et al. [64] evaluated the suitability of 12 commonly used endogenous reference genes to determine which of them was accurate for the normalization of quantification of mRNA expression in human oviducts and normal endometrium. For the endometrium, geNorm™ version 3.5 software analysis demonstrated a reasonable stability of all the genes under study, i.e., enough to be used as HKGs. If a pair of genes were to be applied, these would be *UBC* (ubiquitin C) and *YWHAZ*, yet the best possible combination was obtained with 10 candidate genes. However, the initial recommendation was different with the NormFinder version 0.953 software, namely, to use *UBC* and *ATP5B* (ATP synthase F1 subunit beta) as a pair [64].

A meta-analysis from 2017 on a pool of 646 datasets from 54 different human normal organs/tissues included eight uterine samples but excluded “analyses of individual cell types as the whole organ/tissue includes a vast number of cell types in its structure.” Consequently, the best normalizers for the endometrium were not reported. The eight HKGs proposed for general use in human studies are given in Table 1, with *ACTG1* (actin gamma 1) being the most reliable [65].

Nonetheless, the available evidence can be judged as modest and inconclusive.

## 4. Housekeeping Gene(s) for Studies of Endometrial Cancer

Typical problems associated with many endogenous controls are the presence of genomic DNA contamination, false-positive results generated by genomic pseudogenes, alternatively spliced transcripts, and a high mutation rate [38,66,67,68]. A study specifically focusing on pseudogenes in HKGs found 64 *ACTB* and 67 *GAPDH* pseudogenes in the human genome that were mostly intronless and similar in size to the authentic mRNA. This important work underlined that the design of primers for RT-qPCR needs to avoid mis-priming pseudogenes, and that all primers need to be tested for specificity with both complementary and genomic DNA [69].

One interesting suggestion put forth by a 2002 cancer study was that the nonmetabolic (such as *UBC* or *B2M*) and structural (such as β-tubulin or *ACTB*) HKGs demonstrated lesser level of variation than the metabolic HKGs [22]. We think that this hint is of value and requires careful verification, since metabolic HKGs must demonstrate phenotypic responsiveness to stimuli or inputs from the environment. From the intensive research on solid tumor (that is, non-hematologic and non-lymphoid) malignancies by Bhuva et al., approximately half the stable genes were essential for cell survival, with the remainder being associated with RNA processing, RNA binding, and the spliceosome complex [70]. Another recent study underlined that the most robust reference genes for cancer studies must be irrelevant to malignancy [71].

A TCGA RNA-Seq database pan-cancer study aiming to identify the best reference gene combinations in 12 cancer types found that one of the most frequently used HKGs, *GAPDH*, showed relatively low stability of mRNA levels and did not enter any recommended gene combination. Unfortunately, EC was not included in these analyses [68]. A review on the selection of normalizers for RT-qPCR cancer studies recommended that a combination of *PPIA* and *ACTB*, *HPRT1* (hypoxanthine phosphoribosyltransferase 1), *TBP* (TATA-binding protein), or *GAPDH*, or an appropriate combination of three of these genes, should be employed [72]. For colorectal cancer research, a tandem of *B2M* and *PPIA* as HKGs was recommended [73]. In normal ovarian and ovarian cancer cell lines, the respective genes were *PPIA*, *RPS13* (ribosomal protein S13), and *SDHA* (succinate dehydrogenase complex, subunit A) [74]. The earlier cited Belgian study comparing human malignancies with adjacent healthy tissue found that *mATPsy6* (mitochondrial ATP synthase 6) is suitable as a HKG in kidney, ovarian, and colon cancer studies [53]. An investigation on 13 widely used diverse cancer cell lines and seven finite and immortalized normal cell lines verified the suitability of 12 putative HKGs to conclude, after verification by four algorithmic methods, that the top five reference genes for both cancer cell lines and all cell lines were the same: *HNRNPL* (Heterogeneous Nuclear Ribonucleoprotein L), *IPO8* (importin 8), *PUM1* (pumilio RNA binding family member 1), *SNW1* (SNW domain containing 1), and *CNOT4* (CCR4-NOT transcription complex subunit 4) [75]. Hence, this analysis supported the earlier data from The Human Protein Atlas on the excellent stability of *SNW1* in normal human cell lines [75]. Broad comparative studies on normal human lung cell lines and human lung cancer cell lines under five different experimental conditions established that the four-gene combination of *CIAO1* + *CNOT2* + *CNOT4* + *SNW1* demonstrated the highest stability for the tested culture settings [71]. *CIAO1* is the gene standing behind the Cytosolic Iron-Sulfur Assembly Component 1 protein, whilst *CNOT2* is the CCR4-NOT transcription complex subunit 2 gene. The properly verified stability of *CNOT4* and *SNW1* in these two works [71,75] draws attention and invites further investigation.

Possibly the largest to date study on gene stability in pan-cancer analyzed information derived from 14 independent datasets (with approximately 13,000 samples), including the TCGA and Cancer Cell Line Encyclopedia data. For the prioritization of stable genes in these datasets and simultaneous comparisons against other stable gene lists, expression-based ranks for genes were computed using the product of ranks meta-analysis approach. With such a strategy, a new molecular phenotyping method called “stingscore” was developed. The authors indicated the excellent stability of *HNRNPK* (Heterogeneous Nuclear Ribonucleoprotein K; top ranking), *TARDBP* (TAR DNA binding protein), *CIAO1*, *WDR33* (WD repeat domain 33), *BRAP* (BRCA1 Associated Protein), *NRF1* (Nuclear respiratory factor 1), and *RBM45* (RNA Binding Motif Protein 45) [70].

Initial original research on HKGs in EC studies investigated the suitability of 10 candidate genes in 100 endometrioid histotype samples and 29 normal endometrial tissues. In the evaluation using both the geNorm™ and NormFinder algorithms, the most stably expressed genes turned out to be *HPRT1* and *PPIA*, “to be used alone or better in combination.” However, since *HPRT1* expression showed significant differences between low- and high-grade tumors, the final recommendation was to use *PPIA* as a single reference gene [76]. In a somewhat broader work, Ayakannu et al. evaluated the utility as internal controls in EC of 32 possible genes and concluded that a combination of three, i.e., mitochondrial ribosomal protein L19 (*MRPL19*), *PPIA*, and *IPO8*, is optimal [77]. Consequently, that team used the set of *MRPL19*, *PPIA*, and *IPO8* in their subsequent work [78]. Later, they looked at EC via the prism of the tumor’s histologic types, namely, the endometrioid histotype and the combined serous and carcinosarcoma group [79]. The principal idea was to identify HKGs for Type I (with good prognosis) and Type II (with much worse prognosis) EC. Statistical evaluation by geNorm^PLUS^ version 2.2 with qbase +2 predicted the following optimal normalization in Type I EC: *PSMC4* (Proteasome 26S Subunit, ATPase 4), *PUM1,* and *IPO8* (based on three most stable genes), *ELF1* (E74-like factor 1), *PSMC4*, *PUM1,* and *IPO8* (based on four most stable genes), or *EIF2B1* (eukaryotic translation initiation factor 2B, alpha subunit), *ELF1*, *PSMC4*, *PUM1,* and *IPO8* (based on five most stable genes). For Type II EC, the geometric mean of the reference targets *MRPL19*, *PGK1*, and *PPIA* (based on three most stable genes), *UBC*, *MRPL19*, *PGK1,* and *PPIA* (based on four most stable genes), or *YWHAZ*, *UBC*, *MRPL19*, *PGK1,* and *PPIA* (based on five most stable genes) was predicted as the optimal approach [79]. However, the definitions of Type I and Type II EC have evolved over time, from Bokhman’s straightforward distinction of endometrioid histology tumors in Type I and all other histotypes in Type II disease [80] to a more tumor biology-related division. Bokhman clearly stated that “peculiarities of the tumor (degree of differentiation, depth of invasion, etc.) have not been taken into account” [80], whereas it was gradually acknowledged that Type I represents well-differentiated and moderately differentiated endometrioid tumors with good survival odds, whilst Type II encompasses any poorly differentiated lesion (including the endometrioid histotype), having much poorer survival odds [81,82]. Therefore, the results of this Type I/Type II study need to be applied with prudence, because it used the old distinction. Secondly, the numbers of analyzed cases in particular subgroups were limited (N = 3). Nonetheless, the investigation raised a highly valid problem, i.e., that the research community truly needs EC HKGs that will be universal for any tumor histotype and grade, namely, trusted HKGs of choice, before the final anatomopathological result is known.

For primary and recurrent uterine carcinosarcoma and non-epithelial malignant tumors, e.g., smooth muscle sarcoma and stromal sarcoma, *HPRT1* followed by *UBC* and *HMBS* (hydroxymethylbilane synthase gene) were found to be the most stable HKGs [83]. In studies on ovarian endometrioid adenocarcinoma, *RPLP0* (Ribosomal Protein, large, subunit P0) was validated as a stable HKG [84]. In another study, a comparison of five healthy control ovarian samples with three ovarian endometrioid adenocarcinoma samples demonstrated the least variation in levels of *PPIA*, *RPL37A* (Ribosomal protein L37a), and *RPS17* (Ribosomal protein S17) among the investigated HKGs [85].

In 2022, an international team of researchers recommended for HKG selection a convenient, user-friendly web interface named HouseKeepR (https://exbio.wzw.tum.de/housekeepr; URL accessed on the 20 June 2025) which relies on specified gene expression datasets automatically retrieved from the Gene Expression Omnibus database [86]. HouseKeepR requires only three main input parameters, namely: ‘Tissue type’, ‘Condition’, and ‘Organisms’. Additionally, the number of final HKG candidates can be specified. Table 2 presents the results of our April 2025 searches in the HouseKeepR for best candidates in EC studies. We chose ‘*Homo sapiens*’ as the organism and ‘Carcinoma’ as the condition from the pull-down menus; yet, as tissue type, we went once for ‘Uterus’ and another time for ‘Endometrium’. As can be seen, the searches yielded somewhat different results for this same malignancy, based largely on little known genes, and we were unable to introduce to the tissue type parameter any histotype annotations, such as ‘Endometrioid’, ‘Serous’ or ‘Clear-cell’. A good agreement between tissue types ‘Uterus’ and ‘Endometrium’ was achieved only for the combination of 10 HKGs (with these same nine genes in both categories).

## 5. Our Experience

In 2023, we established transcriptomic profiles of metabolism-related pathways in EC via the exploration of 768 genes using the NanoString nCounter Technology [87]. In a controlled investigation, 57 ECs and 30 normal endometrial specimens were studied using the NanoString Metabolic Panel and further validated by RT-qPCR with a very high similarity. Expression levels of the investigated genes were normalized to the geometric mean of *PPIA* and *ACTB* expressions and reported in line with the Minimum Information for Publication of Quantitative RT-PCR Experiments (MIQEs) guidelines [88]. Relative quantities were corrected for efficiency of amplification, and fold change in gene expression between groups was calculated using the qBase MS Excel VBA for relative quantification using the efficiency of gene-specific amplification [89]. The study successfully identified a substantial deregulation of 11 metabolism-related genes in the EC group. The excellent similarity of the NanoString platform and RT-qPCR results strongly suggested that both HKGs used were suitable for this study’s context.

Our choice to rely on *PPIA* and *ACTB* expression was based on a number of findings. First, we were aware of the many shortcomings of *GAPDH* for this purpose. Second, recommendations from a review focusing on the selection of normalizers for RT-qPCR in cancer were taken into account [72]. Further, *PPIA* expression was found to be more consistent than those of *GAPDH* or *ACTB* in several human organs and tissues studied [13]. In a comparative investigation of seven candidate HKGs in murine kidneys, *PPIA* was identified as the most stable, whereas *GAPDH* was found to be the least stable. In that study, the estimation of the ideal number of genes suggested the use of *PPIA* alone as sufficient, albeit not ideal [90]. In another comparison of 13 candidate HKGs evaluated by three statistical algorithms, i.e., geNorm™, NormFinder and qBasePlus, the pairing of *PPIA* and *B2M* was the most accurate and stable for normalizing RT-qPCR data in colorectal cancer [73]. Somewhat similarly, two gene pairs, i.e., *PPIA* and *HPRT1* and *PPIA* and *IPO8*, were found by geNorm™ and NormFinder, respectively, to be suitable normalizers for studies on non-metastatic and metastatic colon cancer [91]. The second HKG, *ACTB*, was chosen by us based on reassuring results for normal endometrium in Sadek’s study [14].

Yet, in many passages of the present work, criticism of the use of *ACTB* as a HKG is expressed. Similarly, it needs to be kept in mind that *PPIA* may also be a suboptimal reference gene in certain experimental settings. A detailed overview enumerates the varied roles of PPIA in many human diseases, including inflammation and cancer, with its overexpression in the latter and participation in malignant transformation and metastasis [92]. In an in vivo murine cancer model studied by Northern blot analysis, all three HKGs, i.e., *ACTB*, *PPIA*, and *GAPDH*, were found to be significantly upregulated in hepatoma cells compared to adjacent normal liver tissue [93]. A comprehensive review described the known and potential roles of the overexpressed ACTB protein in a broad variety of malignancies [94]. However, *GAPDH*, *ACTB*, and *PPIA* have not been included in the census of human cancer genes [95].

**Figure 1:**
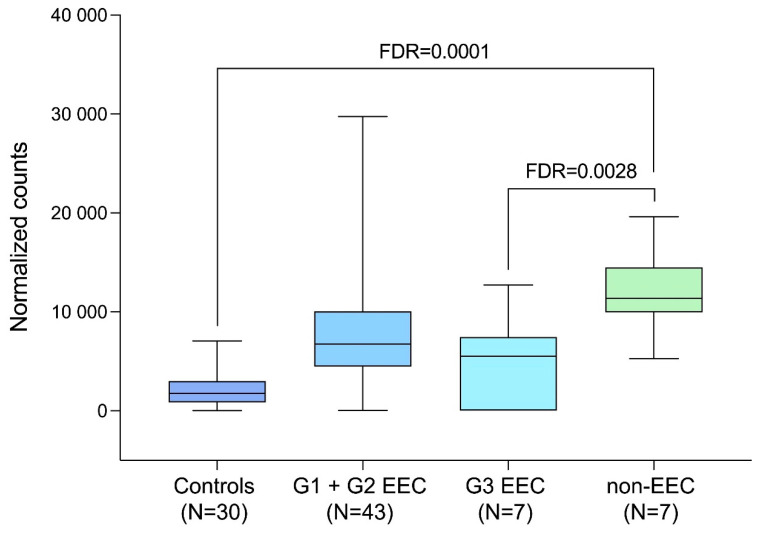
*GAPDH* expression levels in EC. EEC, endometrioid EC; FDR, false discovery rate-adjusted *p*-value; G1, G2, G3—EC histologic grades. Each box-and-whisker plot demonstrates the median and minimal and maximal values.

Table 3 and Figure 1 present our results on *GAPDH* expression in the 2023 EC study. The false discovery rate-adjusted *p*-values were more biologically meaningful here than conventional statistical *p*-values. We found that *GAPDH* overexpression was of borderline significance (*p* = 0.07) when the whole EC group was compared to controls, whereas there was a particularly significant upregulation of *GAPDH* levels observed in the non-endometrioid EC subgroup compared to controls. This overexpression was significantly more pronounced in non-endometrioid than endometrioid ECs. No statistical difference in *GAPDH* expression levels was detected between Type I and Type II cancers. The difference between poorly differentiated endometrioid ECs and non-endometrioid ECs was also not significant, supporting the idea of categorizing poorly differentiated endometrioid tumors as Type II.

## 6. Discrepancies in RT-qPCR Studies on Sex Hormone Receptors in Human Endometrial Cancer Tissue

Some of the authors of this report were involved in a systematic review of data on the expression of the estrogen receptor (ER) and progesterone receptor (PR) isoforms in endometrioid EC. This is a topic of ultimate importance for both a better understanding of EC biology and for tailoring a more individualized treatment of the condition on hormonal grounds. Approved in advance by the Institutional Review Board (Approval No. APK.002.487.2021), the study has been underway since 2021, carried out in line with the PRISMA Group recommendations for systematic reviews [96]. In brief, literature was extensively explored electronically using repeat online database searches from January 1997 to September 2024. Both the Scopus database and MEDLINE^®^ database, searched via the PubMed^®^ search engine, were explored using the Medical Subject Heading-based terms from the National Library of Medicine (Bethesda, MD, USA), alone and in combination. The starting time point was specifically chosen to be 1997, i.e., to be after the publication of an up-to-date review by Nyholm from 1996 [97], at which time knowledge could be summed up as follows: 1) there is a lower content of both ERs and PRs in malignant than in non-malignant endometrium; 2) cytosol receptor positivity for ERs in 79–90% of cases and for PRs in 70–92% of cases was reported in EC; and 3) the content of these receptors decreases with increasing histologic grade. It is noteworthy that knowledge of the existence of ER and PR isoforms was relatively new at that time [98,99,100,101,102,103,104,105,106,107,108,109]. Yet, the implementation of study of these isoforms to EC research followed shortly thereafter, largely through immunohistochemistry (IHC) of protein expression.

The main results derived for RT-qPCR evaluations (alone or combined with other techniques, such as IHC and Western blot) are presented in Table 4 [110,111,112,113,114,115,116,117,118,119,120,121,122,123]. Only 14 reports were identified, and there was a vast discrepancy in the findings. Only one recent report used two HKGs for target gene expression recalculations [123]. Because discordant results have been given, there is little doubt that some of these results are inaccurate in part or in whole. Like for other malignancies, much work on EC has stressed that an inadequate choice of reference gene(s) may obscure genuine changes and/or result in erroneous gene expression data interpretations [72,77,79].

## 7. Necessity of Validation of Housekeeping Genes Before Experimentation

Early research on the topic already underlined the need for the validation of reference RNAs to exclude the possibility that they themselves are expressed at different levels in the studied and control specimens, such as tumor and non-tumor cells [124]. Moreover, malignant tissues often represent genetic instability and heterogeneity [125]. A broad spectrum of data presented in this review testifies to the altered expression of many genes, including *GAPDH*, under different experimental and disease states, and this observation should be widely accepted. Consequently, the suitability of selected reference genes in a given, strictly defined experimental setting must be unconditionally validated prior to each study [23,25,51,126]. Otherwise, systematic errors may be introduced [86]. The recommendation in the MIQE guidelines is that the justification of the choice and number of HKGs should be an essential part of RT-qPCR experiments [88]. Whilst singular reports successfully validating just one HKG exist [53,76,84,90], this is an unlikely situation for the vast majority of studies. The recommendation of Vandesompele et al. from 2002 remains sound: when due consideration is paid to the choice of HKGs, the use of multiple genes for data normalization has the clear potential for superior accuracy [127]. Those authors were able to demonstrate that the conventional use of a single gene for normalization led to large errors (of up to 6.5-fold) in a significant proportion of samples tested [127].

Over time, one can notice the slowly increasing awareness of the problem with arbitrary HKG selections, likely fueled by the MIQE recommendations, i.e., that two or more reference genes should be employed and validated to ensure stable expression across treatment groups for the given experimental setting and sample set [88]. Already in 2002, Tricarico et al. stressed that normalization to a single HKG is inappropriate for human tissue samples [128]. Perhaps for practical reasons of reduced labor, some authors advise that two genes should be taken into account [51,64]. The same opinion on the use of two normalizers was conveyed by the Waxman and Wurmbach study [26]. Sadek et al. recommended that at least two of the three best HKGs should be used for the normalization of target gene(s) expression and presented details of such calculations [14]. From their ovarian cancer studies, Li et al. also advised the use of two or three HKGs [129]. Similarly, Sørby et al. drew a conclusion that two or three reference genes should be used in colon cancer RT-qPCR studies [91]. Others argue that a cohort of at least three control genes needs to be established in advance to experiments [74]. Recent in silico bioinformatics analyses based on data derived from the TCGA database identified *HNRNPL*, *PCBP1* (Poly (RC) Binding Protein 1), and *RER1* (Retention in endoplasmic reticulum sorting receptor 1) as novel pan-cancer reference genes; this was subsequently successfully validated in an array of human cancerous tissues [125]. Unfortunately, the sole systematic review on the effectiveness of use of HKGs found that over the period from 2010 to 2015, the mean number of reference genes utilized in gene expression studies fluctuated around 1.2. Among these, single HKGs were *ACTB* (used in 38% of the studies), *GAPDH* (37%), and 18S rRNA (12%) [130].

Moreover, the study by Jacob et al. documented that reverse transcriptases from different manufactures lead to variations in quantification cycles. Therefore, it may be useful to consider different reverse transcriptases and to test different primer sets prior to undertaking experiments [74]. Similarly, it has been suggested that the selection of different mathematical algorithms (such as geNorm™ of the Ghent University, Ghent, Belgium; NormFinder of Aarhus University, Aarhus, Denmark; BestKeeper^©^; the recently introduced EndoGeneAnalyzer; pair-wise comparative ΔCt method by Vandesompele et al. [127]; or a combination of them) for choosing the most stable HKGs may impact the outcome [74]. Those authors recommend that more than two algorithms should be applied for the final selection of HKGs [74]. The same was observed in earlier studies, where the best candidate gene combination depended on the software used [64,91]. Further, as an alternative to the use of differential expression or variance, mCOPA (modified Cancer Outlier Profile Analysis), an improved algorithm for the analysis of cancer expression data with the identification of both upregulated and downregulated outliers, has been proposed [131]. Although many of these algorithms determine the optimal number of reference genes needed, their initial results may still require recalculations. Specifically, when a different ranking of HKGs by different software applications is present, the PCR cycle threshold coefficient of variation (CtCV%) can be calculated for each of the genes initially deemed to be the most stable as a pair, as recommended by Caradec et al. [25]. As a reflection of the best experimental stability, the lowest value of CtCV% is an indication for final selection.

Table 5 presents a comparison of the methodological principles, strengths, and limitations of the most widely used algorithms.

The updated websites for the current versions of the above calculators are: https://genorm.cmgg.be/; https://biogazelle-qbaseplus.software.informer.com/2.0/; https://www.moma.dk/software/normfinder; https://www.gene-quantification.de/bestkeeper.html; https://exbio.wzw.tum.de/housekeepr; and https://npobioinfo.shinyapps.io/endogeneanalyzer/ (URLs accessed on the 20 June 2025). Other available packages and tools for the analysis of RT-qPCR data include the DeltaCq and CAmpER (Calculation of Amplification Efficiencies for RT-PCR experiments) software. Helpful hints can be found at: https://elearning.vib.be/wp-content/uploads/2020/08/qbase_2018.pdf (accessed on the 12 May 2025).

All consecutive technical steps of RT-qPCR analysis require thorough attention.

## 8. Emerging Strategies and Innovative Tools in HKG Selection

Whereas traditional HKGs have been widely used for RT-qPCR normalization, their suitability for studies investigating non-coding RNA (ncRNA) expression is increasingly being questioned. This is particularly relevant in cancer studies, where the dysregulation of ncRNAs—including microRNAs (miRNAs) and long non-coding RNAs (lncRNAs)—is implicated in tumorigenesis, progression, and therapeutic response. Consequently, efforts have been directed toward identifying more appropriate endogenous controls within the same RNA class, especially for studies focused on ncRNA quantification. Several miRNAs, notably miR-16, miR-423-5p, and U6, have emerged as candidate reference miRNAs in various cancer types, including EC [136,137,138]. These miRNAs are generally expressed at moderate to high levels and are often regarded as being relatively stable across different tissues and experimental conditions. miRNAs currently implicated in EC oncogenesis, usually being up- or downregulated, have been reviewed [139,140,141].

Torres et al. addressed the challenge of selecting appropriate endogenous controls for miRNA expression studies in endometrioid EC tissues. Prior to this study, there was no consensus on suitable reference genes for normalizing miRNA qPCR data in this cancer, and many studies relied on arbitrarily chosen controls. These authors evaluated the expression stability of 12 endogenous candidates in samples from 45 patients, identifying RNU48, U75, and RNU44 as the most stable and therefore reliable for normalization [142].

The HKGs were conventionally selected from a panel of 12 ncRNAs, comprising RNU48, RNU44, U75, RNU6B, U6, U54, RNU38B, U18, U49, miR-26b, miR-92a, and miR-16, all of which have been described as stable in tissues or at least used in EC studies [142]. Nevertheless, their expression showed considerable variability according to some authors and could lead to errors of interpretation and non-reproducibility of the results, according to the histological types concerned. For example, a recent study found that U6 and SNORD48 were not stable reference genes in EC, and therefore, caution should be exercised regarding their use in future studies [143].

Since miRNAs are expressed in tissue-specific patterns, a Japanese study looked at miRNA signatures in blood plasma and endometrial tissue from healthy women and endometrioid EC patients. This research identified eight tumor tissue-associated miRNAs (upregulated: miR-499, miR-135b, and miR-205; downregulated: miR-10b, miR-195, miR-30a-5p, miR-30a-3p, and miR-21). Importantly, two miRNA signatures (miR135b/miR195 and miR135b/miR30a-3p) could accurately distinguish between endometrioid EC and normal endometrial tissue samples. In EC patient plasma, four miRNA alterations were identified (upregulated: miR-135b and miR-205; downregulated: miR-30a-3p and miR-21), and their possible utility as non-invasive markers for early detection of this cancer was suggested [144]. From another paper, serum levels of miR-186, miR-222, and miR-223 were significantly higher in EC patients than in controls, whereas serum miR-204 was significantly lower in EC patients [136].

Despite their potential, the use of miRNAs as reference genes in EC remains preliminary. Current evidence is limited to small cohorts or in vitro studies. None of the aforementioned miRNAs have been universally validated across EC subtypes or sample types (e.g., tissue vs. biofluids). Furthermore, inter-patient variability, tumor heterogeneity, and hormonal regulation of endometrial tissue may affect miRNA expression, necessitating rigorous validation prior to routine use.

Lately, lncRNAs have been increasingly recognized as critical regulators in endometrial carcinogenesis. As such, their use as internal controls for lncRNA-focused studies may offer greater biological relevance than mRNA-based references. Of note, similar to miRNAs, lncRNAs can act as oncogenes or tumor suppressors, depending on intracellular contexts. A listing of upregulated and downregulated lncRNAs in EC was presented in a review [145]. The identification of stably expressed lncRNAs suitable for normalization remains in its infancy, as the inherently low and tissue-specific expression patterns of lncRNAs make their validation as reference genes particularly challenging [145].

Although miRNAs and lncRNAs hold promise as class-specific reference genes in studies focused on ncRNA expression in EC, their validation is currently limited and context-dependent. Future research should aim to systematically evaluate these candidates across large, well-characterized EC cohorts, considering tumor subtype, grade, and sample type. The integration of computational stability assessment tools and biological relevance should guide the selection process. Until such validation is achieved, their use should be approached with caution and, ideally, in conjunction with multiple normalization strategies.

Single-cell RNA sequencing (scRNA-seq) has transformed our understanding of cellular heterogeneity in EC, particularly in terms of revealing the dynamic and cell type-specific nature of gene expression within the tumor microenvironment (TME). These insights have direct implications for the selection and validation of HKGs which are commonly used for data normalization in quantitative expression studies. Recent scRNA-seq studies of primary endometrial tumors showed that genes exhibit substantial variability in expression across the major cellular compartments of the cells involved, such as epithelial tumor cells, stromal fibroblasts, endothelial cells, and infiltrating immune cells [146,147,148]. scRNA-seq data provide compelling evidence that HKG expression in the endometrial TME is not universal, but rather, highly cell type-specific and context-dependent [149]. This reinforces the need for data-driven, cell-specific validation of candidate HKGs, particularly for high-resolution molecular profiling studies. Cellular variability undermines the universal applicability of routinely used HKGs, especially in high-resolution studies or when analyzing discrete cell populations isolated through techniques such as laser-capture microdissection (LCM) or fluorescence-activated cell sorting [150]. The findings are crucial for studies involving spatial transcriptomics or LCM, where normalization must reflect the specific cell type profiled, and for ncRNA investigations, where subtle expression differences may be obscured by inappropriate normalization strategies, thus highlighting the context-dependent expression of HKGs that were merely assumed to be constitutively and uniformly expressed.

Recent methodological advances are revolutionizing how HKGs are identified and validated, ensuring more accurate normalization tailored to the molecular complexity of EC. The integration of artificial intelligence (AI) and machine learning (ML) methods into biomedical research has opened new frontiers in the identification and validation of optimal HKGs, particularly within the complexity of cancer transcriptomes. In EC, where tumor heterogeneity and cellular diversity present significant challenges for accurate gene expression normalization, AI-based models offer a promising solution by leveraging large-scale, multi-dimensional datasets. Recent advances have demonstrated that ML algorithms, such as random forests, support vector machines, and deep neural networks, can analyze high-throughput transcriptomic, epigenomic, and proteomic data to identify context-specific reference gene sets [151]. These tools can move beyond conventional criteria (e.g., low variance, high expression) by considering co-expression patterns, pathway connectivity, cell-type specificity, and sample metadata, including tumor grade, histological subtype, and hormone receptor status. For example, ensemble-based ML classifiers trained on TCGA EC datasets can successfully prioritize genes with stable expression across hundreds of tumor samples, adjusting for batch effects and tumor purity. Further, a multi-layered ML strategy showcases how diverse algorithms can filter and select robust gene sets [152]. A similar approach could be adapted to sift through transcriptomic and multi-omic data to identify stable, context-specific HKG combinations tailored to EC. Moreover, AI models could integrate multi-omics inputs (such as RNA-seq, miRNA-seq, DNA methylation, and proteomics), enabling more robust predictions of HKGs whose expression is not only stable at the transcript level but also resistant to the regulatory perturbations commonly observed in tumorigenesis [153].

Despite the promise of these integrated methods, which offer a holistic view of the biological networks and pathways underpinning cancer, their adoption in experimental practice remains limited due to technical barriers, a lack of user-friendly tools, and insufficient validation across platforms (e.g., RT-qPCR, digital droplet PCR). Nonetheless, AI-driven frameworks are poised to complement or even replace traditional HKG evaluation pipelines by offering customized, data-driven gene selection tailored to the specific experimental context—whether bulk tissue, single-cell data, or liquid biopsies.

## 9. Conclusions

It appears that HKGs have been often chosen arbitrarily rather than systematically. The insufficiently careful selection of *GAPDH* as the sole HKG in many studies conducted so far has contributed to broad discrepancies in published results between scientific centers, even if obtained by the same RT-qPCR technique. The use of *GAPDH* as a HKG should therefore be avoided. Work on *GAPDH* overexpression in EC in relation to overall and relapse-free survival is lacking. The normalization of expression levels of investigated genes with at least two HKGs to the geometric mean of their expression seems to be a rational method for decreasing the internal error and, as such, should be widely applied after careful prior validation. A general consensus on the proper selection of HKGs for research on EC has become of key importance, so that the medical and scientific communities will be in a position to provide more reliable data and to avoid costly and time-consuming RT-qPCR reassessments. Ideally, trusted combinations of HKGs should be universal for any EC histotype and grade, irrespective of the final anatomopathological result.

## Figures and Tables

**Table 1:** The eight meta-analysis-derived HKGs proposed for general use in RT-qPCR studies on human tissues [65]. Full gene names are given as recommended by the Human Genome Organisation’s Gene Nomenclature Committee (www.genenames.org).

Gene Name	Full Gene Name
*ACTG1*	actin gamma 1
*RPS18*	ribosomal protein S18
*POM121C*	POM121 transmembrane nucleoporin C
*MRPL18*	mitochondrial ribosomal protein L18
*TOMM5*	translocase of outer mitochondrial membrane 5
*YTHDF1*	YTH N6-methyladenosine RNA binding protein F1
*TPT1*	tumor protein, translationally-controlled 1
*RPS27*	ribosomal protein S27

**Table 2:** Candidate HKGs for EC studies generated by HouseKeepR [86] based on the selection of either ‘Uterus’ or ‘Endometrium’ as tissue type and as a function of the number of final HKGs. Gene abbreviations: *ATP5PD*, ATP synthase peripheral stalk subunit d; *CD63*, CD63 molecule; *DAD1*, defender against cell death 1; *EEF1G*, eukaryotic translation elongation factor 1 gamma; *EXOSC4*, exosome component 4; *HINT1*, histidine triad nucleotide binding protein 1; *MIDN*, midnolin; *NANS*, N-acetylneuraminate synthase; *RPL19*, ribosomal protein L19; *RPL31*, ribosomal protein L31; *RPLP0*, ribosomal protein lateral stalk subunit P0; *RPS3*, ribosomal protein S3; *RPS5*, ribosomal protein S5; *RPS9*, ribosomal protein S9; *RPS18*, ribosomal protein S18; *TSPO*, translocator protein. Full gene names are given as recommended by the Human Genome Organisation’s Gene Nomenclature Committee (www.genenames.org).

	Selected Tissue Type	
Number of HKGs in Combination	Endometrium	Uterus
2	*RPS3*	*EXOSC4*
	*RPL19*	*MIDN*
3	*EXOSC4*	*RPS9*
	*MIDN*	*RPS18* (ENSG00000223367)
	*NANS*	*RPS18* (ENSG00000226225)
5	*RPLP0*	*RPS9*
	*RPS18* (ENSG00000223367)	*RPS18* (ENSG00000223367)
	*RPS18* (ENSG00000226225)	*RPS18* (ENSG00000226225)
	*TSPO*	*RPL31*
	*HINT1*	*RPS5*
10	*RPS9*	*RPS9*
	*RPS18* (ENSG00000223367)	*RPS18* (ENSG00000223367)
	*RPS18* (ENSG00000226225)	*RPS18* (ENSG00000226225)
	*RPS5*	*RPS5*
	*RPL31*	*RPL31*
	*CD63*	*CD63*
	*ATP5PD*	*ATP5PD*
	*DAD1*	*DAD1*
	*RPS18* (ENSG00000227794)	*RPS18* (ENSG00000227794)
	*EEF1G*	*RPLP0*

**Table 3:** *GAPDH* expression levels in EC. EEC, endometrioid EC; FC, fold change; FDR, false discovery rate; G1, G2, G3—EC histologic grades. For statistical details, see [87]. Numbers of observations are given in parentheses.

*GAPDH* Expression	FC	*p*-Value	FDR-Adjusted *p*-Value
EC (N = 57) vs. Control (N = 30)	4.15	0.000456	0.0700
EEC (N = 50) vs. Control (N = 30)	3.41	0.002482	0.2700
EEC G1 + EEC G2 (N = 43) vs. Control (N = 30)	4.00	0.000508	0.0700
non-EEC (N = 7) vs. Control (N = 30)	10.73	0.000000	0.0001
non-EEC (N = 7) vs. EEC (N = 50)	3.15	0.000393	0.0028
EEC G3 (N = 7) vs. EEC G1 + EEC G2 (N = 43)	4.16	0.249911	1.00
non-EEC (N = 7) vs. EEC G1 + EEC G2 (N = 43)	2.02	0.003000	0.4910
EEC G3 + non-EEC (N = 14) vs. EEC G1 + EEC G2 (N = 43)	1.72	0.239000	1.00
EEC G3 (N = 7) vs. non-EEC (N = 7)	12.13	0.077883	1.00

**Table 4:** A summary of the pertinent literature on the expression of estrogen receptor (ER) and progesterone receptor (PR) isoforms in endometrial cancer (EC) [110,111,112,113,114,115,116,117,118,119,120,121,122,123]. G1, G2, G3—EC histologic grades; FIGO, The International Federation of Gynecology and Obstetrics; GAPDH, glyceraldehyde-3-phosphate dehydrogenase; HKG, housekeeping gene; HPRT1, hypoxanthine phosphoribosyltransferase 1; IHC, immunohistochemistry; mRNA, messenger RNA; ND, not detected; NS, not studied; POLR2A, RNA polymerase II subunit A; RT-PCR, real time-polymerase chain reaction (not always described as quantitative); YWHAZ, Tyrosine 3-monooxygenase/tryptophan 5-monooxygenase activation protein zeta; **↓**—reduced gene expression; **↑**—gene overexpression. For clarity, studies that were non-human, solely on cell lines, competitive RT-PCR-Southern blot, or carried out without the involvement of RT-PCR were excluded. The nomenclature is given as provided in the cited references.

Authors, Publication Year	RT-PCR HKG(s)	Evaluation Method(s)	ERα	ERß	PRA	PRB
Saegusa & Okayasu, 2000 [110]	*β-actin*	RT-PCR and IHC	Wild type transcripts detected in 41/48 (85.4%) of tumors; **↓** *ERα* with increasing grade; varied immunointensity and distribution of immuno-positive cells by IHC	Wild type expression observed in 22/34 (64.7%) of tumors; weak immune-reactivity sporadically observed in a few cases of tumor epithelial cells; no relation with histologic grade by IHC	No distinction between *PRA* and *PRB* made; *PR* mRNA positive in 47/48 (97.9%) of tumors, **↓** *PR* with increasing grade; by IHC, varied immunoreactivity; immunoreactivity scores for PR higher than for ERα	
Utsunomiya et al., 2000 [111]	*β-actin*	mRNA in situ hybridization, RT-PCR and IHC	45 tumors were studied with no controls; *ERα* mRNA detected in 36/45 (80.0%) of cases	*ERβ* mRNA detected in 16/45 (35.6%) of cases; among the 16 *ERβ* positive cases, 15 were also *ERα* positive; thus, *ERβ* is coexpressed with *ERα*	Solely PR labeling index was given	
Jazaeri et al., 2001 [112]	*β2-microglobulin*	RT-PCR and Western blot	**↓** *ERα* mRNA expression in EC (N = 7)	*ERα* mRNA expression exceeds that of *ERβ*	*PRA* mRNA levels inferred by subtracting *PRB* mRNA values from total PR (N = 8)	A relative abundance of *PRB* mRN
Kershah et al., 2004 [113]	*β-actin*	RT-PCR and Western blot	**↑** in the expression of *ERα* mRNA in EC, yet not at the protein level; no difference between endometrioid and non-endometrioid tumors in mRNA expression	*ERβ* NS	No distinction between PRA and PRB made; no significant changes in expression levels between normal premenopausal endometrium (N = 26) and EC (N = 30 of varied histology)	
Skrzypczak et al., 2004 [114]	*GAPDH*	RT-PCR	**↓** *ERα* in EC (N = 19) compared to normal endometrium (N = 21)	Expression of total *ERβ*, *ERβ1*, *ERβ2*, *ERβ2Δ5*, *ERβ3*, *ERβ4*, and *ERβ5* were studied; **↓** of *ERβ2Δ5*, no expression of *ERβ3*, very low expression of *ERβ4*, and **↑** of *ERβ5* in EC were reported	No distinction between PRA and PRB made; no significant changes in expression levels between normal endometrium (N = 21) and EC (N = 19)	
Pathirage et al., 2006 [115]	*18S rRNA*	RT-PCR	**↑** *ERα* in postmenopausal G1 EC (N = 7) compared to higher grade tumors (N = 10) and normal premenopausal endometrium (N = 20)	A trend for G1 tumors to express higher *ERβ* levels than G2 and -3 tumors	NS	NS
Chakravarty et al., 2007 [116]	*β-actin*	RT-PCR, Western blotting, and IHC	Studied yet data not reported	Low levels of expression of *ERβ1* in EC (N = 26); **↓** ERβ2/βcx at the protein level when compared to normal proliferative endometrium (N = 22), yet no statistical difference at the transcript level; a significant **↓** *ERβ2/βcx* in G2 tumors compared to G1 tumors	Studied yet data not reported; *PR* expression correlated with *ERα* expression, no correlation of *PR* with *ERβ1* or -*β2/βcx* expression	
Šmuc & Lanišnik Rižner, 2009 [117]	*PPIA* for RT-PCR, β-actin for Western blotting	RT-PCR, Western blot and IHC	**↓** *ERα* in EC (N = 16) compared to adjacent normal endometrium	**↓** *ERβ* in EC (N = 16) compared to adjacent normal endometrium	PRA: NS; PR-AB studied instead (N = 16) and found **↓** in EC	**↑** expression of PRB at the protein level in EC (N = 16) in Western blot
Häring et al., 2012 [118]	*β-actin*	RT-PCR, cell culture, and Western blot	**↓** *ERα* in G3 EC (N = 15) compared to normal pre- and postmenopausal endometrium (N = 28) or to G1 (N = 15) or G2 (N = 16) tumors	Compared to normal endometrium (N = 28), no difference in expression for *ERβ1* and -*2*; **↑** *ERβ5*, **↑** *ERβ∆1*, **↑** *ERβ∆2/3* and **↓** *ERβ∆4* in cancer (N = 46); expression of *ERβ1* and *ERβ2* strongly correlated with *ERα* expression	No distinction between PRA and PRB; data only reported as a strong correlation of *ERα* transcript levels with *PR* expression	
Jarzabek et al., 2013 [119]	*18S rRNA*	RT-PCR and IHC	Significantly decreased mRNA and protein expression levels in EC (N = 48) as compared to normal endometrium (N = 15); a positive correlation between ERα and ERβ	Significantly decreased mRNA and protein expression levels in EC (N = 48) as compared to normal endometrium (N = 15); negative correlations between levels of ERα and ERβ transcripts and depth of myoinvasion; a negative correlation of ERβ mRNA expression with FIGO staging	NS	NS
Wik et al., 2013 [120]	*GAPDH*	RT-PCR, IHC, single-nucleotide polymorphism array, and Sanger sequencing	ERα negativity: in 19/76 (25%) of primary investigation cases, in 35/155 (22.6%) of prospective validation cohort cases, and in 68/286 (23.8%) of retrospective validation cohort cases; over 50% of ERα-negative tumors were G3; low ERα was strongly associated with poor patient survival	NS	NS	NS
Kamal et al., 2016 [121]	*YWHAZ*	RT-PCR and IHC	A reduction in stromal expression of *ERα* in EC when compared with healthy premenopausal controls (N = 28)	*ERβ* was the predominant steroid receptor expressed in both low-grade- (N = 37) and high-grade (N = 48) EC; a general reduction in the expression of steroid receptors in high-grade EC compared with healthy premenopausal tissue	No distinction between PRA and PRB; a reduction in stromal expression of *PR* in EC when compared with healthy premenopausal controls	
Kasoha et al., 2020 [122]	*β-actin*	RT-PCR on prospective samples and IHC on retrospective samples	No expression difference between EC (N = 16) and normal endometrium (N = 6)	No expression difference between EC (N = 17) and normal endometrium (N = 6); no immunostaining differences for ERβ1 and ERβ5 either; significantly lower immunopositivity for ERβ2 in EC	NS	NS
Hojnik et al. 2023 [123]	*HPRT1* and *POLR2A* for RT-PCR, GAPDH for Western blot	RT-PCR, Western blot and IHC	Decreased mRNA expression in 44 tissue pairs of EC and adjacent normal endometrium; a significant correlation of *ERα* and *ERβ* expression at the mRNA level	Decreased mRNA expression in EC in 34 tissue pairs; no significant changes in the *ERα/ERβ* expression ratio in EC	NS	NS

**Table 5:** A comparison of commonly used tools for HKG stability analysis in RT-qPCR. CV, coefficient of variation; Ct, cycle threshold; SD, standard deviation.

Tool	Methodological Principle(s)	Advantage(s)	Limitations	Reference
geNorm™	Pairwise variation of gene expression (M value); optimal gene number (V value)	Simple; indicates required number of reference genes	Assumes no co-regulation; favors similarly expressed genes	[127]
NormFinder	Model-based estimation of intra- and inter-group variation	Accounts for experimental groups; robust to co-regulation	Requires grouping information; less intuitive	[132]
BestKeeper	Ct-based SD, CV, and correlation analysis	Easy to use; works directly with raw data	Assumes normality; sensitive to outliers	[133]
RefFinder	Integrates geNorm™, NormFinder, BestKeeper, and ΔCt method	Combines results for consensus ranking; user-friendly	Limited customization; dependent on included algorithms’ assumptions	[134]
EndoGeneAnalyzer	Combines multiple statistical approaches (NormFinder, SD, correlation analysis); integrates stability assessment with normalization	Raw qRT-PCR Ct data	Stability ranking of HKGs; outlier detection; impact assessment on target gene normalization	[135]

## Data Availability

The original data on *GAPDH* expression presented in this review are included in Figure 1 and Table 3. Data that support the findings of this study have been deposited in the Gene Expression Omnibus (GEO) database (Accession Number: GSE196033). Further inquiries can be directed to the corresponding author.
